# Workplace bullying and psychological distress of employees across socioeconomic strata: a cross-sectional study

**DOI:** 10.1186/s12889-019-6859-1

**Published:** 2019-06-13

**Authors:** Caryn Mei Hsien Chan, Jyh Eiin Wong, Lena Lay Ling Yeap, Lei Hum Wee, Nor Aini Jamil, Yogarabindranath Swarna Nantha

**Affiliations:** 1 Faculty of Health Sciences, National University of Malaysia, Kuala Lumpur, Malaysia; 2 Primary Care Department, Tuanku Jaafar Hospital, Negeri Sembilan, Malaysia; 3 Stats Consulting Malaysia, Kuala Lumpur, Malaysia

**Keywords:** Workplace bullying, Psychological distress, K6, Mental health, Employee health, Socioeconomic status, Vulnerable populations

## Abstract

**Background:**

1Little is known of the extent of workplace bullying in Malaysia, despite its growing recognition worldwide as a serious public health issue in the workplace. Workplace bullying is linked to stress-related health issues, as well as socioeconomic consequences which may include absenteeism due to sick days and unemployment. We sought to examine the prevalence of workplace bullying and its association with socioeconomic factors and psychological distress in a large observational study of Malaysian employees.

**Methods:**

This study employed cross-sectional, self-reported survey methodology. We used the 6-item Kessler screening scale (K6) to assess psychological distress (cutoff score ≥ 13, range 0–24, with higher scores indicating greater psychological distress). Participants self-reported their perceptions of whether they had been bullied at work and how frequently this occurred. A multivariate logistic regression was conducted with ever bullying and never bullying as dichotomous categories.

**Results:**

There were a total of 5235 participants (62.3% female). Participant ages ranged from 18 to 85, mean ± standard deviation (M ± SD): 33.88 ± 8.83. A total of 2045 (39.1%) participants reported ever being bullied. Of these, 731 (14.0%) reported being subject to at least occasional bullying, while another 194 (3.7%) reported it as a common occurrence. Across all income strata, mean scores for psychological distress were significantly higher for ever bullied employees (M ± SD: 8.69 ± 4.83) compared to those never bullied (M ± SD: 5.75 ± 4.49). Regression analysis indicated significant associations (*p* < 0.001) between workplace bullying with being female (Adjusted OR (aOR) = 1.27, 95% CI 1.12–1.44), higher individual income levels of between RM4,000 to RM7,999 (aOR =1.24, 95% CI 1.06–1.45) and RM8,000 and above (aOR = 1.31, 95% CI 1.10–1.56), and psychological distress (aOR = 1.15, 95% CI 1.13–1.16).

**Conclusions:**

More than one in three employees reported having experienced workplace bullying, which was found to be specifically associated with being female, drawing a higher income, and greater psychological distress. In general, low individual income was associated with greater psychological distress. However, higher income employees were far more likely to report experiencing workplace bullying. Findings from this study offer relevant insight into the associations between socioeconomic status and psychological distress in workplace bullying.

## Background

Workplace bullying is gaining recognition as a serious public health issue. Bullying among the working population poses a widespread threat to employee health [1], both physical and psychological, with direct socioeconomic consequences [2, 3].

While the forms of work bullying are myriad, the outcomes associated with bullying are singularly negative. In particular, workplace bullying carries implications for mental health. Past research demonstrates that work bullying has an adverse impact on the mental wellbeing of not just employees who are bullied, but has been shown to demoralise and affect witnesses and bystanders as well [4].

The psychological consequences of workplace bullying, particularly in the context of added vulnerabilities associated with low income is akin to a double setback for employees already struggling on a day to day basis with the challenges and pressures of work demands and responsibilities.

It has been documented in the organisational literature that economically and/or socially disadvantaged groups in the workplace are more vulnerable towards being victimized as a result of workplace bullying [1]. Individuals from lower income groups, as well as employees who have ever experienced workplace bullying, have been separately shown to be at heightened risk of poorer mental health.

This is important given that socioeconomic disparities are a main determinant and contributor of persisting health inequity. The association between workplace bullying and psychosocial adversities may be mediated by socioeconomic status, which, in developed countries [5], is postulated to aggregate in low socioeconomic sectors of the population.

Little is known of the extent of workplace bullying in Malaysia [5–7] despite its growing acknowledgement worldwide as a grave public health issue in the working population. To this end, we sought to examine the prevalence of workplace bullying and its association with socioeconomic factors and psychological distress among Malaysian employees. We posit the hypothesis that workplace bullying and psychological distress are likely to aggregate in low socioeconomic status groups.

## Methods

### Study design and participants

The Malaysia’s Healthiest Workplace study by AIA Bhd., a leading life insurance group, builds on the success of Britain’s Healthiest Workplace [8]. An online questionnaire survey was conducted in a work-based sample of 5369 Malaysian employees aged 18–85 years in Malaysia. This study employed cross-sectional, internet-based self-reported survey methodology.

Employers (one representative from the Human Resource Department from each organisation) and all eligible employees of interested organisations were approached via an email blast to participate in the survey. The link to the survey was sent along with information to employees after the organisation agreed participate. Participating organisations received a comprehensive report (Organisational Health Report). Employees who completed the survey received a Personal Health Report.

A total of 47 corporate companies in Malaysia participated in the study between 18th of May to 18th of July 2017.

### Survey questionnaire

The questionnaire survey covers comprehensive dimension of employment, occupation, company size, education, household income, and subjective social status, with the following variables of interest examined:

#### Work bullying

Participants self-reported their perceptions of whether they had been bullied at work and how frequently this occurred with the statement: ‘I am subject to bullying at work’, with the five following response options: ‘never’, ‘seldom’ ‘sometimes’, ‘often’, ‘always’. A dichotomous category split was imposed on the bullying data for purpose of analysis. Respondents who indicated ‘never’ were classified as ‘never bullied’, while those who indicated ‘seldom’ ‘sometimes’, ‘often’, ‘always’ were categorised as ‘ever bullied’.

#### Psychological distress

We used the 6-item Kessler screening scale (K6) to assess psychological distress. The K6 has a score range of 0–24, with higher scores indicating greater psychological distress. A cutoff score ≥ 13 was used to detect nonspecific psychological distress, a threshold which has been used to screen for severe mental illness estimated to afflict about 6% of US adults [9]. Alternately, a cutoff score ≥ 5 has been proposed as the optimal lower threshold cut-point indicative of moderate mental distress [10].

#### Socioeconomic status

Socioeconomic status was assessed on the basis of individual monthly income self-reported by the participants to the closest RM999 bracket. We categorised individual income according to the distribution which best approximates the widely used bottom 40% (B40), middle 40% (M40) and top 20% (T20) strata following the Khazanah Research Institute’s household income classification for Malaysia [11].

### Covariates

Demographic characteristics (age, gender, educational level and marital status) were assessed with a general checklist.

### Statistical analyses

Descriptive statistics are presented for all demographic characteristics. Differences between groups were analysed using univariate Chi-Square (X^2^) and t-test. Comparison of independent means for ever bullied and never bullied employees were run using a two-sample t-test. Mean differences in psychological distress scores between the never bullied and ever bullied groups were tested. A multivariate logistic regression was conducted with ever bullying and never bullying as dichotomous categories, with individual income and psychological distress as independent variables using the enter method. We used the standard method of entry by simultaneously entering all independent variables and covariates into the equation at the same time. The adjusted odds ratio (OR) statistic with 95% confidence interval (CI) was calculated to analyse the association between work bullying, psychological distress and socioeconomic strata. Further subset analysis was conducted using work bullying, psychological distress and individual income by strata in a 3 by 3 table. Statistical comparisons were two-sided, using *p* < 0.05 as the level of significance. All analyses were performed using the Statistical Package for the Social Sciences [12].

## Results

The initial sample consisted of 5369 individuals. After excluding non-Malaysian participants, the final sample comprised *N* = 5235 participants from 47 organisations in Malaysia. A female preponderance was observed (*n* = 3259, 62.3%). Participant age ranged from 18 to 85, mean ± SD: 33.88 ± 8.83. The mean employment length (M ± SD) 6.17 ± 6.94 years. A total of 2045 (39.1%) participants reported ever being bullied at the workplace (Table 1). In terms of work bullying frequency, *n* = 1120 (21.4%) reported it as ‘seldom’ occurring, while *n* = 731 (14.0%) indicated ‘sometimes’, *n* = 145 (2.8%) as ‘often’, and *n* = 49 (0.9%) as ‘always’ (Table 1).

**Table 1:** Characteristics of employees from 47 organisations in Malaysia (*N* = 5235)

Characteristic	Never bullied *n* = 3190	Ever bullied *n* = 2045	*X* ^2^ ^a^
Gender			0.001
Male	1286 (40.3)	690 (33.7)	
Female	1904 (59.7)	1355 (66.3)	
Age (years)			0.001
18–24	435 (13.6)	269 (13.2)	
25–34	1389 (43.5)	1000 (48.9)	
35–44	901 (28.2)	562 (27.5)	
45–54	368 (11.5)	178 (8.7)	
55–64	94 (2.9)	35 (1.7)	
≥ 65	3 (0.1)	1 (0.0)	
Ethnicity			0.001
Malay	1280 (40.1)	555 (27.1)	
Chinese	1299 (40.7)	1051 (51.4)	
Indian	515 (16.1)	375 (18.3)	
Other	96 (3.1)	64 (3.1)	
Marital status			0.001
Single	1389 (43.5)	983 (48.1)	
Married	1638 (51.3)	934 (45.7)	
Separated/ Divorced	62 (1.9)	39 (1.9)	
Widowed	23 (0.7)	8 (0.4)	
Prefer not to say	78 (2.4)	81 (4.0)	
Educational attainment			0.135
No formal education, primary, lower & upper secondary	270 (8.5)	163 (8.0)	
Post secondary	578 (18.1)	328 (16.0)	
Undergraduate degree	1769 (55.5)	1195 (58.4)	
Postgraduate degree	593 (18.0)	359 (17.6)	
Occupational group			0.224
Manager	995 (31.2)	587 (28.7)	
Professional	896 (28.1)	602 (29.4)	
Technician or junior professional	300 (9.4)	191 (9.3)	
Clerical support worker	372 (11.7)	223 (10.9)	
Service worker	32 (1.0)	23 (1.1)	
Sales worker	110 (3.4)	80 (3.9)	
Skilled agricultural/ forestry/ fishery worker	1 (0.0)	0 (0.0)	
Plant and machine operator or assembler	4 (0.1)	0 (0.0)	
Elementary occupations	1 (0.0)	1 (0.0)	
Other	265 (8.3)	163 (8.0)	
Don’t know	43 (1.3)	37 (1.8)	
Prefer not to answer	171 (5.4)	138 (6.7)	
Work irregular hours			0.005
No	2558 (80.2)	1574 (77.0)	
Yes	632 (19.8)	471 (23.0)	
Psychological distress (K6)			0.001
K6 score of 0 to 12	2930 (63.9)	1657 (36.1)	
K6 score of 13 and above	260 (40.1)	388 (59.9)	
Individual income			0.426
≤ RM 3999	1136 (35.6)	720 (35.2)	
RM 4000 to RM 7999	923 (28.9)	625 (30.6)	
RM 8000 and above	1131 (35.5)	700 (34.2)	
Financial concerns			0.001
No	568 (17.8)	488 (23.9)	
Yes, a little	1261 (39.5)	638 (31.2)	
Yes, a lot	1361 (42.7)	919 (44.9)	

*Note*: Percentages by column

^a^Pearson’s Chi-square test, 2 tailed

Multivariate logistic regression analysis indicated significant associations (*p* < 0.001) between workplace bullying with being female (Adjusted OR (aOR) = 1.27, 95% CI 1.12–1.44), higher monthly individual income levels of between RM4,000 to RM7,999 (aOR =1.24, 95% CI 1.06–1.45) and RM8,000 and above (aOR = 1.31, 95% CI 1.10–1.56), and psychological distress (aOR = 1.15, 95% CI 1.13–1.16) (Table 2).

**Table 2:** Multivariate logistic regression analyses examining predictors of work bullying among employees (*N* = 5235)

Predictors	*p-value*	Adjusted OR	95% CI
Gender			
Male (Ref.)	–	1.00	–
Female	0.001	1.27	1.12–1.44
Age	0.216	1.01	1.00–1.02
Marital status			
Single	0.395	0.89	0.68–1.17
Married	0.246	0.86	0.66–1.11
Separated/ Divorced/ Widowed/ Other (Ref.)	–	1.00	–
Educational attainment			
No formal education, primary, secondary level	0.223	0.86	0.67–1.10
Post secondary	0.538	0.93	0.74–1.17
Undergraduate degree	0.480	0.91	0.70–1.18
Postgraduate degree (Ref.)	–	1.00	–
Individual income			
≤ RM 3999 (Ref.)	–	1.00	–
RM 4000 to RM 7999	0.008	1.24	1.06–1.45
RM 8000 and above	0.002	1.31	1.10–1.56
Psychological distress (K6)	0.001	1.15	1.13–1.16

Employees from the lowest income strata had the highest levels of psychological distress. Approximately half (50.3%) of all employees who reported significant psychological stress (K6 scores ≥13) were from the lowest income bracket (≤RM3,999 monthly). Within the lowest income group (≤RM 3999), mean scores for psychological distress were significantly higher for ever bullied employees (M ± SD: 9.93 ± 4.76) compared to those never bullied (M ± SD: 6.78 ± 4.69). This was also true across all socioeconomic strata (Table 2).

Within-category analyses indicate that there was a significant mean difference in psychological distress scores between never bullied and ever bullied employees from lowest to highest individual income categories of 3.15, 2.66 and 2.93 respectively (*p* = 0.001). By economic strata, the largest mean difference between never bullied and ever bullied employees were those from the lowest individual income bracket (≤RM 3999) (Table 3).

**Table 3:** Means and standard deviations for psychological distress scores stratified by work bullying status and individual income among employees (*N* = 5235)

Variable (score range)	Psychological distress					
	Never bullied *n* = 3190		Ever bullied *n* = 2045		Total *n* = 5235	
	M ± SD	N (%)	M ± SD	N (%)	M ± SD	N (%)
Individual income						
≤ RM 3999^a^	6.78 ± 4.69	1136 (35.6)	9.93 ± 4.76	720 (35.2)	8.00 ± 4.97	1856 (35.5)
RM 4000 to RM 7999^b^	5.83 ± 4.65	923 (28.9)	8.49 ± 4.87	625 (30.5)	6.90 ± 4.91	1548 (29.6)
RM 8000 and above^c^	4.66 ± 3.86	1131 (35.5)	7.59 ± 4.56	700 (34.2)	5.78 ± 4.37	1831 (35.0)
Overall (all income levels)	5.75 ± 4.49	n = 3190	8.69 ± 4.83	n = 2045	6.90 ± 4.84	N = 5235

*Note*: Percentages by column

^a,b,c^: all *p*-values are significant at a 0.05 level

## Discussion

This study represents a rare attempt to examine workplace bullying and the mental health status of employees with careful socioeconomic differential in the Malaysian setting. While the psychological consequences of work bullying on health have received extensive attention, much less consideration has been accorded to identifying psychosocial risk factors, specifically how socioeconomic differences may mitigate work bullying outcomes.

We sought to examine the prevalence of workplace bullying and its association with individual-level socioeconomic factors and psychological distress among Malaysian employees. Specifically, we explored the hypothesis that workplace bullying and psychological distress are likely to aggregate in low socioeconomic status groups.

A high prevalence rate for workplace bullying was found among Malaysian employees. More than one in every three employees (39.1%) reported ever having experienced workplace bullying. Almost half of all employees who reported ever being bullied indicated being subject to at least occasional bullying (sometimes, often and always). The high prevalence of workplace bullying in this setting is particularly notable. Put into perspective, this rate is more than double the reported global prevalence rates of work bullying of approximately 15% in developed and developing nations [13]. This finding is concerning as our prevalence of 39.1% is more than double the global rate of 15% [13], particularly so when compared against the lower prevalence (9.0–15%) typically found in Asian countries including Japan [1]. The rate found in our sample is also far higher than the prevalence of 18.1% found for self-labelling studies without a given definition of work bullying [13].

As we had initially hypothesized that workplace bullying was likely more prevalent among employees from low socioeconomic backgrounds, it was an unexpected finding that workplace bullying in our study was found to be specifically associated with drawing a higher income. Being female and greater psychological distress was also linked to ever being bullied at work. However, our findings did support the notion that low individual income was associated with greater psychological distress. Thus several psychosocial risk factors for workplace bullying in this setting were identified: being female, drawing a higher income, and greater psychological distress.

In general, employees from the lowest individual income bracket reported the highest levels of psychological distress, with slightly over half all employees who reported significant psychological distress earning monthly individual income of ≤RM3,999. On closer examination however, regression analyses revealed that higher income employees were more likely to report ever being bullied at the workplace. These findings refute the hypothesis that workplace bullying and psychological distress both aggregate in low socioeconomic status groups, and of itself these findings are unsurprising for a developing economy of a middle-resource country [14–16].

These findings suggest that sociodemographic factors appear to play a role in workplace bullying. Indeed, across a wide income discrepancy seen amongst our sample of employees in this study, workplace bullying appears to cluster among employees with higher income brackets. We should however not allow this to obscure the fact that bullying occurs across all economic strata. Income levels per se, therefore, may not be a clear indicator for risk of workplace bullying even where economic disparities are also evident.

In our study, there were clear gender differences in terms of work bullying prevalence, with a higher proportion of female employees who reported ever being bullied at work, compared to their male counterparts. This is at odds with recent evidence in the literature [17, 18] which argue that work bullying is a gender-neutral phenomenon. Our findings ally with the general dictum drawn from the bulk of evidence and overall consensus of studies which consider work bullying to be a gendered issue [19].

It remains unclear whether female employees are more likely than males to experience or to report work bullying. Past research however have shown that women at the workplace may be more vulnerable to workplace bullying [20], and together with employees with mental health difficulties and employees from lower income brackets, form a vulnerable population whom may be susceptible to bullying at work and enduring poorer mental health.

It is also important to remember that work bullying affects not just females, but males as well. Gender does not mitigate levels of psychological distress experienced by bullied employees. This is why we may need to reduce stigma around men’s mental health and encourage them to seek help when they are struggling [21] with not just issues such as work bullying, but for symptoms of anxiety and depression in general.

In both genders separately, there was a positive association between workplace bullying and high psychological distress, and between high psychological distress and low socioeconomic status. Specifically, across all socioeconomic strata, levels of psychological distress were found to be higher among employees who had ever experienced bullying at the workplace compared to employees who reported no prior experience of work bullying.

It is also worth highlighting that the impact of workplace bullying on psychological distress was found to be greater among employees with higher individual income. Scant attention to psychological wellbeing, which is fairly typical in an Asian setting due to the stigma attached to mental illnesses and which discourages help-seeking [22], may partly explain the high prevalence of psychological distress in this setting [23, 24]. The underlying mechanism might also be socioeconomic [14, 16].

Individuals who are bullied at work and experience psychological distress as a consequence are at greater risk of having or exacerbating poor mental health [25], and possibly not being likely to seek help and support for their distress due to the double setback of (1) not wanting to be stigmatised by seeking psychological support, therefore not using available support services if provided by their company, and (2) being less likely than high income individuals to be able to afford out of pocket mental health treatment.

While it has been well documented that workplace bullying can lead to psychological distress, past research also show that socially disadvantaged groups in the working population, such as employees with poor mental health (individuals facing psychological adversities) [25] or from lower income groups, are at heightened risk of being victimized by workplace bullying [15, 16].

Findings from this study may help shed some insight into certain groups of employees at higher risk or vulnerability, which may have a higher vulnerability towards work bullying – in this case, women, lower socioeconomic status individuals and employees facing psychological adversities.

### Strengths and limitations

To the best of our information, this is the first study which reports prevalence of workplace bullying and its association with socioeconomic factors and psychological distress in a large sample of Malaysian employees recruited from multiple organisations.

It should be noted that work bullying was measured by self-report without a specific given definition of work bullying. Findings should therefore be interpreted with the reminder that any causality cannot be determined due to the cross-sectional nature of our study. We cannot determine causality, thereby rendering it impossible to determine whether (i) the high psychological distress was a result of work bullying, or (i) employees with high psychological distress were more likely to be bullied at work. Respondent socioeconomic status was classified by individual and not household income, which would have been ideal. In addition, the exact participation rate for the study cannot be determined due to the fact that we cannot hazard an estimate of the number of employees who actually received or read the invitation email, nor can we rule out selection bias.

### Recommendations

Our findings suggests that exposure to several risks (being female, presence of psychological distress and drawing a low income) is likely to influence the overall likelihood and consequence of experiencing workplace bullying among employees in an additive or possibly even interactive way. Although research so far has failed to establish such exacerbated effects, more research is needed on these issues.

The prevalence of psychological distress was highest among individuals who have ever experienced bullying at work and low-income earners, although higher income and greater psychological distress was found to be associated with workplace bullying. This lends credence to the association between workplace bullying and psychological distress being different at the economic level. Although psychological distress in general may be consistently associated with low socioeconomic status, psychological distress associated with workplace bullying appear to be tied to higher income employees.

Socioeconomic status provides little guidance for identifying which employees are at risk of workplace bullying. All employers and employees, not just those with greater socioeconomic deprivation, should be targeted to reduce the adverse effects of bullying as economically disadvantaged individuals may be far less likely to seek needed support.

Future studies are needed to test whether socioeconomic differences are a consistent determinant of workplace bullying and psychological distress. Further longitudinal studies are warranted to confirm the presented results and explain the relationship between workplace bullying, psychological distress and socioeconomic status.

## Conclusion

Workplace bullying is an issue which requires greater focus and priority as its adverse association on mental health means workplace bullying will continue to be an ongoing challenge within the workplace. Findings from this study offer relevant insight into employee wellbeing in a developing country. Findings additionally highlight the vulnerability of employees who are bullied at work, working women and the economically disadvantaged for whom we need to improve their status at the workplace. Our findings carry implications for employee health and workplace health planning to mitigate these disparities.

## Abbreviations

aOR: Adjusted odd ratio
CI: Confidence interval
K6: 6-item Kessler screening scale
SD: Standard deviation
